# Death is associated to the type of drug-eluting stent in patients with left ventricular dysfunction and elevated natriuretic peptide levels

**DOI:** 10.1038/s41598-021-81569-x

**Published:** 2021-01-28

**Authors:** Christian Roth, Clemens Gangl, Walter S. Speidl, Georg Goliasch, Matthias Schneider, Daniel Dalos, Rudolf Berger

**Affiliations:** 1 Department of Internal Medicine II, Cardiology, Medical University of Vienna, Vienna, Austria; 2 Department of Internal Medicine I, Cardiology and Nephrology, Hospital of St. John of God, Eisenstadt, Austria

**Keywords:** Interventional cardiology, Outcomes research

## Abstract

As advanced heart failure (HF) with elevated NT-proBNP is characterized by an activated coagulation system, coronary events clinically noticed as sudden or HF death may be more common after treatment with first- compared to newer-generation DES. Our study evaluates (1) if patients with left ventricular dysfunction (LVSD) who underwent percutaneous coronary intervention have a better survival with first- or newer-generation DES, and (2) if the survival benefit is predicted by NT-proBNP. Our observational study evaluated patients with LVSD who were registered in the coronary catheter laboratory database of the Medical University of Vienna. Multivariate Cox regression analyses tested an interaction in the risk of death between those with lower or elevated NT-proBNP levels and the stent-generation. The relative risk of newer- compared to first-generation DES as reference was calculated for patients with low and elevated NT-proBNP levels. In 340 patients (178 newer- and 162 first-generation DES) stent-generation and NT-proBNP were independent predictors of death. When the stent-generation*NTproBNP interaction was forced into a Cox regression model, this term independently predicted death. The relative risk of first- compared to newer-generation DES was similar in patients with lower NT-proBNP (HR 1.02, 95% CI 0.95–1.10, p = 0.560), but was higher in patients with elevated NT-proBNP (HR 1.06, 95% CI 1.01–1.10, p = 0.020). Death is associated to stent-generation. NT-proBNP is a predictor for the stent generation used: elevated levels demonstrated a higher mortality risk when using first- compared to newer-generation DES, while lower levels showed a similar risk when using either DES-generation.

## Introduction

First-generation DES, especially sirolimus-eluting-stents (SES) and paclitaxel-eluting-stents (PES), demonstrated clear superiority in preventing restenosis and the need for repeat revascularization compared to bare-metal stents. Nevertheless, remote follow-up data revealed higher rates of late and very late thrombotic events. Newer-generation DES were designed to overcome these long-term safety issues and, indeed, reduced stent thrombosis in the early and long term. However, these improvements in stent design like thinner stent-struts and improved tissue-compatibility of the polymer^1^ could not be translated into benefits regarding survival or target lesion revascularization compared to first-generation DES in patients with stable coronary artery disease^2,3^*.*

Heart failure (HF) based on left ventricular systolic dysfunction (LVSD) is associated with increased risk of stent thrombosis. This systemic disorder is accompanied by a prothrombotic environment due to an upregulation of prothrombotic and downregulation of antithrombotic factors^4^. This prothrombotic shift seems to be more pronounced in more severe HF. Natriuretic peptides vary from normal levels to excessive elevation in patients with moderate to severe LVSD and, thereby, indicate different stages of disease^5^. Accordingly increasing levels of natriuretic peptides correlate with an increasing activation of the coagulation system as displayed by elevated D-dimer and von Willebrand Factor (vWF)^6^. In various clinical settings, higher levels of N-terminal proB-type Natriuretic Peptide (NT-proBNP) were related to thrombotic findings like thrombosis of transcatheter heart valves^7^ or spontaneous echo contrast as precursor for left atrial thrombosis^8^. In two pooled contemporary HF trials high NT-proBNP levels identified a subset of HF patients without atrial fibrillation at a high risk of stroke^9^.

In most patients with ischemic heart failure the mode of death is clinically described as sudden cardiac or pump failure death. Especially when death occurs outside of the hospital, information is frequently lacking on prodromal symptoms; an electrocardiogram and cardiac biomarkers are rarely obtained^10^. However, autopsy studies reported coronary pathologies like plaque ruptures, intracoronary thrombi, and acute myocardial infarction to be frequent findings^11,12^. These coronary events are often clinically not recognized, but may trigger sudden or pump failure death. Due to the procoagulant state patients with ischemic cardiomyopathy and severe HF have an increased risk for stent-thrombosis and myocardial infarction^13^. Our study analyses if elevated NT-proBNP levels indicate worse outcome in patients with moderate to severe LVSD and coronary multivessel disease who were revascularized using first- compared to newer-generation DES.

## Methods

### Study population and data collection

We included adult patients from the coronary catheter laboratory database of the Medical University of Vienna (CCLD-MUW) between the years 2004 and 2012 in this observational cohort study. All patients suffered from significant CAD and LVSD. The definition of significant CAD was a stenosis of the left main stem ≥ 50% or major coronary artery ≥ 70% or 50–70% with objective evidence of myocardial ischemia and had to be present in at least one major coronary artery. For inclusion into the study left ventricular ejection fraction assessed by echocardiography (Simpson biplane) had to be below or equal to 40%^14^. In addition, NT-proBNP at time of hospital admission had to be available. At this time-point, all baseline characteristics were evaluated. The PCI was performed by experienced interventionists during the hospital stay of the diagnostic coronary angiography. Patients who underwent coronary artery bypass grafting (CABG) were excluded from analysis. Only adult patients with informed consent were included. CCLD-MUW provided the following data: baseline characteristics, co-morbidities, angiographic characteristics, blood results and echocardiographic parameter. The patients observational period started with initial coronary angiography and ended at the due day, which was the 31st of December 2014. All patients in < were managed according to the relevant heart failure guidelines of the European Society of Cardiology in one out of five heart failure clinics in Vienna. As there are no services such as telemedicine or homecare available for these patients in Vienna, additional multidisciplinary management was not possible for any of these patients. The survival status at due day was retrieved from the official Austrian death registry. The study was approved by the Ethics Committee of the Medical University of Vienna and is in line with the Declaration of Helsinki.

### Statistical analysis

Continuous variables were presented as median and interquartile range (IQR), categorical variables as counts or percentages. Continuous variables were presented by using an unpaired T-test, categorical data were presented by using crosstabs and chi-square test. Due to the relatively small study population and the exclusion of a significant number of unmatched patients with loss of according information when using propensity score matching we preferred covariate adjustment^15^. Cox proportional hazards regression models were applied to identify independent predictors of death from any cause. These models included all variables that were univariate predictors of death except those which were available in less than 50% of patients (Fibrinogen, D-Dimer, Total cholesterol, C-reactive protein). NT-proBNP levels were not normally distributed, therefore log transformed NT-proBNP levels were taken for continuous analysis within the Cox proportional hazards regression models. No methods came into operation to fill-up missing data or adjust the model for the presence of missing values^16^.

Clinically relevant covariates were implicated into following multivariable models.

- Model A: To clarify if DES-generation (first or newer) is associated with death, death was adjusted by age, sex, rhythm, prior myocardial infarction, diabetes mellitus (DM), estimated glomerular filtration rate (Cockcroft-Gault formula; eGFR), Canadian Cardiovascular Society (CCS) class, New York Heart Association (NYHA) class, number of coronary vessels diseased, smoker, cerebrovascular and/or peripheral vascular disease, ejection fraction (EF) and DES-generation.
- Model B: To confirm an association between death and levels of NT-proBNP, death was adjusted by age, sex, rhythm, prior myocardial infarction, DM, eGFR, CCS class, NYHA class, number of coronary vessels diseased, smoker, cerebrovascular and/or peripheral vascular disease, EF and NT-proBNP.
- Model C: To identify an interaction between DES-generation and NT-proBNP, death was adjusted by age, sex, rhythm, prior myocardial infarction, DM, eGFR, CCS class, NYHA class, number of coronary vessels diseased, smoker, cerebrovascular and/or peripheral vascular disease, EF, NT-proBNP, DES-generation, and NT-proBNP*DES-generation.

In order to present the results of model C more clearly, we added two multivariate models.

- Model D: To answer if DES-generation is associated with death in patients with above median NT-proBNP, death was adjusted by age, sex, smoker, cerebrovascular and/or peripheral vascular disease, and DES-generation.
- Model E: To clarify no association between DES-generation and death in patients with below median NT-proBNP, death was adjusted by age, sex, DM, CCS class, number of coronary vessels diseased, cerebrovascular and/or peripheral vascular disease, and DES-generation.

All variables, which were significant in these COX proportional hazards regression models are shown in Forest plots. With 152 deaths in the whole study population and 110 deaths in patients with NT-proBNP above median, the analyses stick to the rule of thumb with a minimum of ten outcome events per predictor variable. With 42 deaths this target is not exactly met in patients with NT-proBNP below median (seven variables), however, recent reports estimated this rule as too conservative^17^. To stratify NT-proBNP into two groups of lower and elevated values, the median NT-proBNP of 2927 pg/ml (IQR 1282/8524) was used. As there was an even number of patients in this study, the median of NT-proBNP values was calculated by taking the average of the two middlemost numbers. Contingency tables were used to compare the relative risk of PCI with first- and newer-generation DES for patients with low and elevated NT-proBNP levels. The SPSS 24.0 (IBM Corp.) was used for all statistical analyses^16^.

## Results

**Figure 1:**
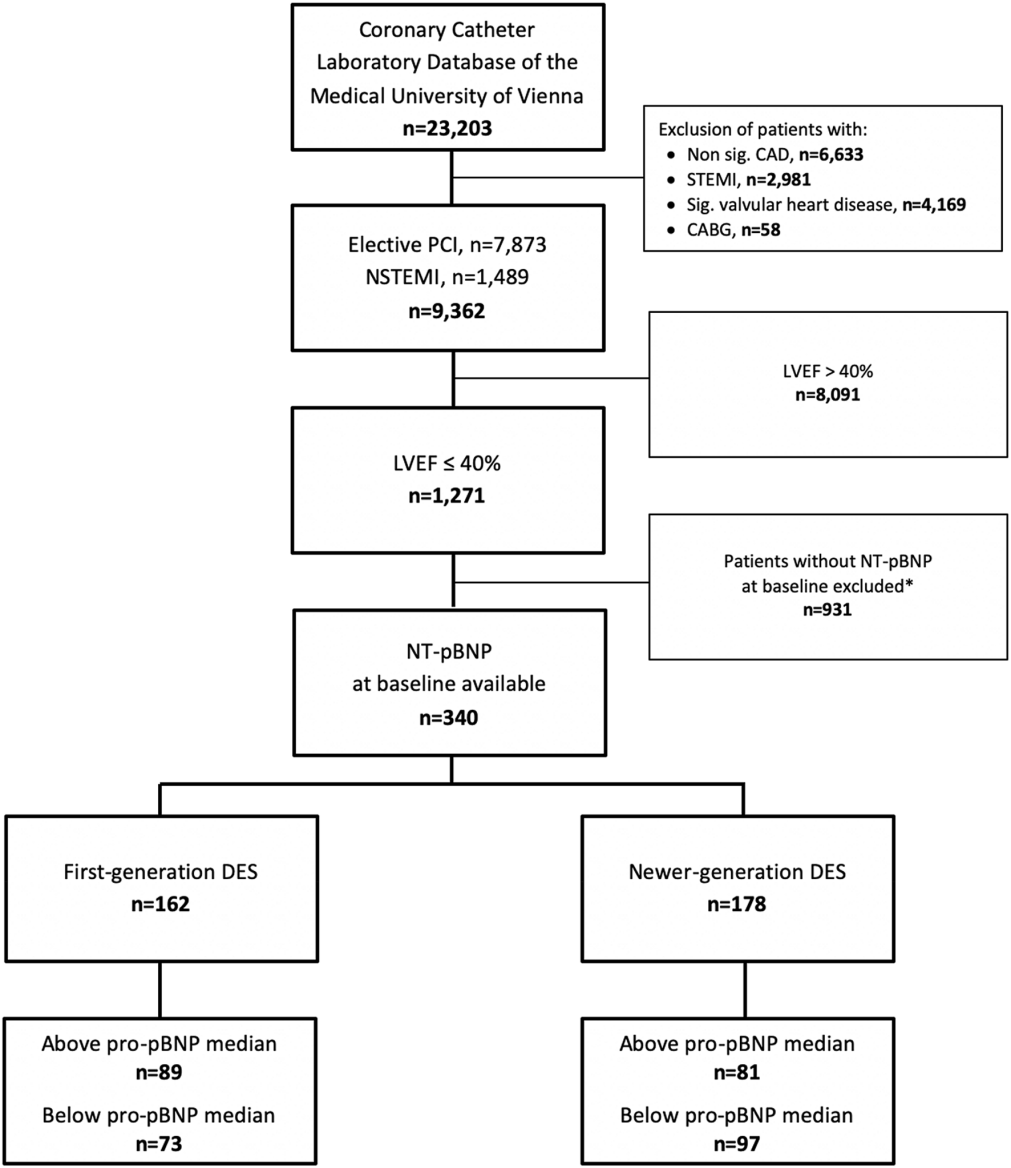
Flow chart. NT-pBNP median (2927 pg/ml, IQR 1282/8524). *CABG* coronary artery bypass graft, *CAD* coronary artery disease, *DES* drug-eluting stent, *LVEF* left ventricle ejection fraction, *NSTEMI* non-ST-segment elevation myocardial infarction, *NT-proBNP* N-terminal proB-type Natriuretic Peptide, *PCI* percutaneous coronary intervention, *STEMI* ST-segment elevation myocardial infarction. *NT-proBNP testing was routinely performed in only one of the three cardiology wards of the general hospital due to different standard laboratory analyses upon admission.

This study included 340 patients with CAD and moderate to severe LVSD from the CCLD-MUW. Figure 1 shows a detailed flow chart of the study population. Patients characteristics of the whole population are presented in Tables 1 and 2. When using a modified definition of ischemic etiology all 340 patients either had a prior myocardial infarction (40%) or a coronary multivessel disease (two or more vessels with ≥ 75% stenosis–67%) or a ≥ 75% stenosis of the left main coronary artery/proximal LAD (18%) or any combination^18^. Almost half of the patients (n = 164) underwent at least one or more coronary angiographies during the observation period. Table 3 displays the last available treatment of these patients. This pharmacologic treatment is well in line with that described in a large prospective, observational European registry^19^. Details regarding cause of death are given in Table 4. During the observation period 152 patients (45%) died with a median observation time of 1507 days (IQR 810/2471).

**Table 1:** Demographics, comorbidities and clinical presentation of all patients, subgroups according to DES-generation and subgroups with above and below NT-pBNP median (2927 pg/ml, IQR 1282/8524).

	All n = 340	DES-generation		P-value	Above NT-pBNP median (> 2927 pg/ml) n = 170	Below NT-pBNP median (< 2927 pg/ml) n = 170	P-value
		First-generation n = 162	Newer-generation n = 178				
Age (years); median (IQR)	66 (58/75)*	66 (58/74)*	66 (48/76)*	0.362	70 (63/79)*	64 (54/71)*	< 0.001
**Gender**				0.179			0.892
Female; n (%)	67 (20)	27 (17)	40 (22)	–	33 (19)	34 (20)	–
Male; n (%)	273 (80)	135 (83)	138 (78)	–	137 (81)	136 (80)	–
Body-mass-index (kg/m^2^); median (IQR)	27 (25/30)	27 (24/30)	27 (25/30)	0.560	27 (24/29)	28 (25/30)	0.054
Hypertension; n (%)	261 (77)	122 (75)	139 (78)	0.544	134 (79)	127 (75)	0.369
Hyperlipidemia; n (%)	254 (74)	123 (76)^‡^	131 (74)	0.622	127 (75)	127 (75)	1.000
Diabetes mellitus; n (%)	133 (39)*	65 (40)	68 (38)*	0.717	76 (45)	57 (34)^†^	0.035
**Smoker**	^†^	^‡^	^‡^	0.624	^‡^		0.095
Current; n (%)	102 (30)	49 (30)	53 (30)	–	45 (27)	57 (34)	–
Previous; n (%)	66 (19)	28 (17)	38 (21)	–	29 (17)	37 (22)	–
Family history; n (%)	67 (20)	35 (22)	32 (18)	0.401	28 (17)	39 (23)	0.134
Cerebrovascular and/or peripheral vascular disease; n (%)	119 (35)*	65 (41)^‡^	54 (31)^†^	0.083	84 (50)^‡^	35 (20)^‡^	< 0.001
Prior myocardial infarction; n (%)	135 (40)^‡^	72 (44)	63 (35)^‡^	0.088	70 (41)	65 (38)	0.579
Prior PCI; n (%)	80 (24)	33 (20)	47 (26)	0.190	32 (19)	48 (28)	0.041
Prior CABG; n (%)	53 (16)	25 (15)	28 (16)	0.940	26 (15)	27 (16)	0.881
**NYHA class**	^†^	^‡^		0.094		^‡^	0.307
Class I; n (%)	79 (23)	30 (19)	49 (28)	–	38 (22)	41 (24)	–
Class II; n (%)	51 (15)	22 (13)	29 (16)	–	24 (14)	27 (16)	–
Class III; n (%)	146 (43)	80 (49)	66 (37)	–	81 (48)	65 (38)	–
Class IV; n (%)	64 (19)	30 (19)	34 (19)	–	27 (16)	37 (22)	–
**CCS class**	*	*		0.275		*	0.080
Class I; n (%)	61 (18)	34 (21)	27 (15)	–	35 (21)	26 (15)	–
Class II; n (%)	69 (20)	30 (19)	39 (22)	–	32 (19)	37 (22)	–
Class III; n (%)	112 (33)	57 (35)	55 (31)	–	63 (37)	49 (29)	–
Class IV; n (%)	98 (29)	41 (25)	57 (32)	–	40 (23)	58 (34)	–
Elective/NSTEMI				0.760			0.380
Elective; n (%)	144 (42)	70 (43)	74 (42)	–	68 (40)	76 (45)	–
NSTEMI; n (%)	196 (58)	92 (57)	104 (58)	–	102 (60)	94 (55)	–

*CABG* coronary artery bypass graft, *CCS* Canadian Cardiovascular Society, *DES* drug-eluting stent, *IQR* interquartile range, *NSTEMI* non-ST-segment elevation myocardial infarction, *NT-proBNP* N-terminal proB-type Natriuretic Peptide, *NYHA* New York Heart Association, *PCI* percutaneous coronary intervention.

Univariate predictor for death: *p ≤ 0.001; ^†^p ≤ 0.010; ^‡^p ≤ 0.050.

**Table 2:** Baseline therapy and electrocardiographic, echocardiographic and coronary angiographic results of all patients, subgroups according to DES-generation and subgroups with above and below NT-pBNP median (2927 pg/ml, IQR 1282/8524).

	All n = 340	DES-generation		P-value	Above NT-pBNP median (> 2,927 pg/ml) n = 170	Below NT-pBNP median (< 2,927 pg/ml) n = 170	P-value
		First-generation n = 162	Newer-generation n = 178				
ACE-inhibitor/ARB; n (%)	340 (100)	162 (100)	178 (100)	–	170 (100)	170 (100)	–
Beta-bocker; n (%)	271 (80)	133 (82)	138 (76)	0.295	135 (80)	136 (80)	0.893
Aldosteron; n (%)	172 (51)	84 (52)	88 (49)	0.657	94 (55)	78 (46)	0.083
Aspirin; n (%)	340 (100)	162 (100)	178 (100)	–	170 (100)	170 (100)	–
Clopidogrel; n (%)	315 (93)	159 (98)	156 (88)	< 0.001	161 (95)	154 (91)	0.146
Ticagrelor; n (%)	25 (7)	3 (2)	22 (12)	< 0.001	9 (5)	16 (9)	0.146
Pacemaker, n (%)	28 (8)	17 (11)	11 (6)	0.148	17 (10)	11 (7)	0.237
ICD, n (%)	42 (12)	15 (9)	27 (15)	0.098	16 (9)	26 (15)	0.099
NT-proBNP (pg/ml) (n); median (IQR)	2,927 (1,282/8,524)*	2,776 (1,304/7,832)*	3,178 (1,177/9,214)*	0.425	8,498 (5,187/15,187) *	1,286 (558/2,059) ^‡^	< 0.001
Total cholesterol (mg/dl); median (IQR)	175 (144/213)^†^	173 (146/208)*	177 (143/218)	0.520	167 (136/207)	181 (152/220)	0.002
HDL (mg/dl); median (IQR)	42 (35/50)	42 (34/50)	42 (35/49)	0.871	40 (35/47)	43 (34/53)	0.014
LDL (mg/dl); median (IQR)	89 (69/113)	87 (68/114)	89 (74/112)	0.705	87 (65/106)	95 (74/117)	0.716
Creatine (mg/dl); median (IQR)	1.12 (0.94/1.46)*	1.08 (0.93/1.36)	1.22 (1.00/1.54)^†^	0.018	1.32 (1.06/1.75)	1.03 (0.89/1.23)	< 0.001
eGFR, median (IQR)	58 (48/68)^†^	59 (48/68)^‡^	58 (46/69)^‡^	0.703	54 (41/65)	64 (54/72)	< 0.001
Sodium (mmol/l); median (IQR)	139 (137/141)	139 (137/140)	139 (137/141)	0.523	138 (136/141)	139 (137/140)	0.951
Potassium (mmol/l); median (IQR)	4.1 (3.8/4.4)	4.1 (3.8/4.3)	4.1 (3.8/4.4)	0.502	4.1 (3.7/4.5)	4.0 (3.8/4.3)	0.179
CRP (mg/dl); median (IQR)	1.41 (0.45/4.79)^‡^	1.08 (0.40/4.33)	1.94 (0.60/6.31)	0.488	2.43 (0.86/8.85)	0.81 (0.34/2.27)	< 0.001
D-Dimer (µg/ml); median (IQR)	0.95 (0.41/1.83)	0.75 (0.39/1.41)	1.13 (0.61/2.10)	0.996	1.32 (0.70/2.15)	0.51 (0.34/1.02)^†^	0.015
Fibrinogen (mg/dl); median (IQR)	481 (394/580)*	468 (393/559)^‡^	502 (404/585)^†^	0.482	515 (424/643)	449 (378/537)^‡^	< 0.001
Prothrombine time (%); median (IQR)	90 (75/106)	89 (75/104)	91 (75/107)	0.618	87 (71/103)	92 (77/107)	0.022
aPTT, (s); median (IQR)	38 (34/44)	38 (34/46)	37 (33/42)	0.031	39 (34/46)	37 (33/43)	0.425
INR; median (IQR)	1.2 (1.1/1.4)	1.2 (1.1/1.4)	1.3 (1.1/1.4)	0.708	1.3 (1.2/1.4)	1.2 (1.1/1.4)	0.035
Heart rate (bpm); median (IQR)	77 (66/88)	76 (66/91)	78 (66/85)†	0.061	80 (68/90)	75 (65/85)	0.006
**Rhythm**				0.221			0.067
Sinus rhythm; n (%)	292 (86)	139 (86)	153 (86)	–	138 (81)	154 (91)	–
Atrial fibrilation; n (%)	31 (9)	13 (8)	18 (10)	–	20 (12)	11 (6)	–
Other; n (%)	17 (5)	10 (6)	7 (4)	–	12 (7)	5 (3)	–
Ejection fraction; median (IQR)	33 (25/37) ^‡^	34 (29/38)	32 (22/37)	0.079	32 (24/37)	34 (29/38)	0.039
**No. of coronary vessels diseased**	*	*		0.045		‡	0.045
1 VD; n (%)	112 (33)	46 (28)	66 (37)	–	47 (28)	65 (38)	–
2 VD; n (%)	72 (21)	43 (27)	29 (16)	–	34 (20)	38 (22)	–
3 VD; n (%)	156 (46)	73 (45)	83 (47)	–	89 (52)	67 (40)	–
**DES-generation**	*			–	*	*	0.082
First-generation; n (%)	162 (48)	–	–	–	89 (52)	73 (43)	
Newer-generation; n (%)	178 (52)	–	–	–	81 (48)	97 (57)	

*ACE* angiotensin-converting-enzyme, *aPTT* activated partial thromboplastin time, *ARB* angiotensin-receptor blocker, *CABG* coronary artery bypass graft, *CRP* C-reactive protein, *DES* drug-eluting stent, *eGFR* estimated glomerular filtration rate (Cockcroft-Gault formula), *HDL* high density lipoprotein, *ICD* implantable cardioverter-defibrillator, *INR* International Normalized Ratio, *IQR* interquartile range, *LDL* low density lipoprotein, *NSTEMI* non-ST-segment elevation myocardial infarction, *NT-proBNP* N-terminal proB-type Natriuretic Peptide, *PCI* percutaneous coronary intervention, *VD* vessel disease.

Univariate predictor for death: *p ≤ 0.001; ^†^p ≤ 0.010; ^‡^p ≤ 0.050.

**Table 3:** Traetmernt at baseline and follow-up.

	Baseline (n = 164)	Follow-up (n = 164)
ACE-inhibitor/ARB; n (%)	164 (100)	164 (100)
Beta-bocker; n (%)	133 (81)	146 (89)
Aldosteron; n (%)	87 (53)	116 (71)
Aspirin; n (%)	164 (100)	164 (100)
Clopidogrel; n (%)	157 (96)	120 (73)
Ticagrelor; n (%)	7 (4)	2 (1)
Pacemaker, n (%)	10 (6)	11 (7)
ICD, n (%)	15 (9)	23 (14)

**Table 4:** Cause of death.

All	All n = 340	First-generation n = 162	Newer-generation n = 178
Follow-up duration (days) (IQR)	1507 (810/2471)	2148 (839/3159)	1331 (805/1859)
**Total death; n (%)**	152 (45)	100 (62)	52 (29)
Sudden death; n (%)	14 (4)	8 (5)	6 (3)
Heart failure; n (%)	85 (25)	61 (38)	24 (13)
Myocardial infarction; n (%)	29 (9)	19 (12)	10 (6)
Other; n (%)	24 (7)	12 (7)	12 (7)
Alive; n (%)	188 (55)	62 (38)	126 (71)

Above NT-pBNP median (> 2927 pg/ml)	All n = 170	First-generation n = 89	Newer-generation n = 81
Follow-up duration (days) (IQR)	1033 (543/2099)	1077 (511/2259)	916 (597/1790)
**Total death; n (%)**	110 (65)	74 (83)	36 (44)
Sudden death; n (%)	12 (7)	7 (8)	5 (6)
Heart failure; n (%)	62 (37)	45 (50)	17 (21)
Myocardial infarction; n (%)	20 (12)	14 (16)	6 (7)
Other; n (%)	16 (9)	8 (9)	8 (10)
Alive; n (%)	60 (35)	15 (17)	45 (56)

Below NT-pBNP median (< 2927 pg/ml)	All n = 170	First-generation n = 73	Newer-generation n = 97
Follow-up duration (days) (IQR)	1851 (1182/2816)	2908 (2002/3496)	1546 (1028/1928)
**Total death; n (%)**	42 (25)	26 (36)	16 (17)
Sudden death; n (%)	2 (1)	1 (1)	1 (1)
Heart failure; n (%)	23 (14)	16 (22)	7 (8)
Myocardial infarction; n (%)	9 (5)	5 (7)	4 (4)
Other; n (%)	8 (5)	4 (6)	4 (4)
Alive; n (%)	128 (75)	47 (64)	81 (83)

*CABG* coronary artery bypass graft, *DES* drug-eluting stent, *NT-proBNP* N-terminal proB-type natriuretic peptide, *PCI* percutaneous coronary intervention.

### Results of the Cox proportional hazards regression models

Predictors of death as calculated in different Cox proportional hazards regression models are shown in Table 5.

- In model A DES-generation was an independent predictor of death in the whole study population including 340 patients (162 first-generation and 178 newer-generation). The baseline characteristics of patients treated with first- and newer-generation DES are presented in Tables 1 and 2.
- Model B demonstrated the distinguished significance of NT-proBNP as a prognostic marker in patients with reduced LVF: NT-proBNP and age were the strongest independent predictors of death. To compare baseline characteristics of patients with different degrees of disease severity we dichotomized the patient population according to the NTproBNP median. The baseline characteristics of patients with NT-proBNP levels below and above the median (2927 pg/ml) are presented in Tables 1 and 2.
- Model C showed a significant interaction between DES-generation and NT-proBNP: DES-generation was significantly associated with death, but only in patients with NT-proBNP levels above median.

**Table 5:** Predictors of death in Cox proportional hazards regression analyses.

	p-value	Hazard ratio	Lower confidence limits	Upper confidence limits
**Model A**				
Age	< 0.001	1.05	1.02	1.07
DES-generation	0.049	1.06	1.00	1.11
Estimated glomerular filtration rate	0.054	0.98	0.97	1.00
Cerebrovascular and/or peripheral vascular disease	0.140	1.41	0.89	2.21
NYHA	0.181	1.18	0.93	1.49
Diabetes mellitus	0.231	1.32	0.84	2.07
Rhythm	0.388	1.17	0.82	1.65
Ejection fraction	0.440	0.99	0.97	1.01
Number of coronary vessels diseased	0.542	1.11	0.82	1.45
Prior myocardial infarction	0.616	0.90	0.58	1.38
Smoker	0.648	0.94	0.71	1.24
Gender	0.751	0.92	0.53	1.58
CCS	0.977	1.00	0.81	1.25
**B Model**				
NT-proBNP	< 0.001	1.38	1.24	1.53
Age	0.002	1.04	1.01	1.06
NYHA	0.076	1.23	0.98	1.55
Number of coronary vessels diseased	0.085	1.30	0.97	1.74
Estimated glomerular filtration rate	0.121	0.99	0.97	1.00
Cerebrovascular and/or peripheral vascular disease	0.296	1.27	0.81	2.00
CCS	0.617	0.94	0.75	1.19
Ejection fraction	0.639	0.99	0.97	1.02
Prior myocardial infarction	0.664	0.91	0.59	1.40
Diabetes mellitus	0.676	1.11	0.69	1.76
Gender	0.720	0.90	0.51	1.60
Smoker	0.860	1.03	0.77	1.37
Rhythm	0.968	0.99	0.68	1.44
**C Model**				
NT-proBNP*DES-generation	< 0.001	1.08	1.04	1.12
DES-generation	0.001	0.59	0.42	0.80
Age	0.003	1.04	1.01	1.06
NT-proBNP	0.009	1.24	1.06	1.45
Number of coronary vessels diseased	0.026	1.42	1.04	1.94
NYHA	0.113	1.21	0.96	1.53
Rhythm	0.190	1.29	0.88	1.89
Cerebrovascular and/or peripheral vascular disease	0.231	1.32	0.84	2.09
Diabetes mellitus	0.412	1.22	0.76	1.96
Prior myocardial infarction	0.626	0.90	0.59	1.40
CCS	0.716	1.04	0.83	1.31
Ejection fraction	0.720	1.01	0.98	1.03
Estimated glomerular filtration rate	0.865	0.10	0.98	1.02
Gender	0.963	1.01	0.57	1.79
Smoker	0.992	1.00	0.75	1.34
**D Model**				
Age	0.002	1.04	1.01	1.06
DES-generation	0.020	1.06	1.01	1.10
Cerebrovascular and/or peripheral vascular disease	0.044	1.48	1.01	2.17
Smoker	0.557	0.92	0.71	1.20
Gender	0.588	0.87	0.53	1.43
**E Model**				
Age	0.002	1.05	1.02	1.08
Diabetes mellitus	0.048	1.93	1.01	3.68
CCS	0.103	0.79	0.59	1.05
Cerebrovascular and/or peripheral vascular disease	0.152	1.63	0.83	3.20
DES-generation	0.560	1.02	0.95	1.10
Number of coronary vessels diseased	0.926	0.98	0.67	1.43
Gender	0.964	1.02	0.48	2.17

(Model A) DES generation was an independent predictor of death in the entire study population. (Model B) NT-proBNP was an independent predictor of death. (Model C) A significant interaction between NT-proBNP and DES generation. (Model D) Association between DES generation and death in patients with above median NT-proBNP. (Model E) No association between the DES generation and death in patients with below median NT-proBNP.

*CCS* Canadian Cardiovascular Society, *NYHA* New York Heart Association.

In order to present the results of model C more clearly, we stratified patients according to their NT-proBNP median and assessed the impact of the DES-generation on the risk of death in different stages of HF: Out of 170 patients with NT-proBNP levels above median 89 patients underwent PCI with first-generation DES and 81 patients underwent PCI with newer-generation DES. Out of 170 patients with NT-proBNP levels below median 73 patients underwent PCI with first-generation DES and 97 patients underwent PCI with newer-generation DES.

- Model D demonstrated an association between death and DES-generation in patients with NT-proBNP levels above median.
- In model E, which focused on patients with NT-proBNP levels below median, this association could not be confirmed.

### Relative risk of death of DES-generation in patients with NT-proBNP levels above and below median

The relative risk of death in patients who underwent PCI with first-generation DES compared to newer-generation DES was similar in patients with lower NT-proBNP levels (p = 0.558), but was significantly increased in patients with elevated NT-proBNP levels (p = 0.015).

## Discussion

Death is associated to stent-generation. NT-proBNP is a predictor for the stent generation used: elevated levels demonstrated a higher mortality risk when using first- compared to newer-generation DES, while lower levels showed a similar risk when using either DES-generation.

### Stent thrombosis as trigger for a survival difference

In comparison with first-generation DES, second-generation DES have maintained the low restenosis rates of first-generation devices, but additionally reduced rates of stent thrombosis. However, this difference could not be translated in a survival benefit^2,3^. In contrast, our analysis shows, that patients with elevated NT-proBNP levels had a higher mortality risk when using first- compared with newer-generation DES. This finding may result from the fact that heart failure patients are characterized by a prothrombotic activation of the coagulation system, thereby significantly augmenting the risk of stent thrombosis. However, acute coronary events such as stent thrombosis or myocardial infarction are often masked as sudden or pump failure death in these patients.

### Prothrombotic state and inflammation

Chronic HF is associated by a prothrombotic state due to an upregulation of prothrombotic factors and a downregulation of antithrombotic factors. This prothrombotic shift^20^ is based on an increased platelet activity as well as elevated levels of blood coagulation markers (fibrinopeptide A, plasma fibrinogen) and fibrinolytic factors (D-Dimer)^4,20^. Recently, a British study reported an association between NT-proBNP and plasma coagulation factors in older men^6^. In accordance our data clearly demonstrated higher levels of fibrinogen and D-dimer in more severe HF patients defined by higher NT-proBNP levels compared to patients with less severe HF (Table 2). Therefore, our findings support evidence that the extent of the prothrombotic shift in these patients correlates to the severity of HF as described by the activation of the natriuretic peptide system. However, these markers of coagulation and thrombosis did not interact with NT-proBNP*DES-generation. This finding is not surprising since these factors play a subordinate role in the development of arterial thrombi: activated plasma coagulation factors enhance venous blood clotting, whereas platelet activation increases the risk of arterial stent-thrombosis. Unfortunately, platelet function is not tested in patients undergoing PCI in our daily routine and not available for evaluation.

### Mode of death

In patients with ischemic cardiomyopathy autopsy studies described coronary pathologies like plaque ruptures, intracoronary thrombi, and acute myocardial infarction to be frequent findings^11,12^. These coronary events are often clinically not recognized, but may trigger sudden cardiac death or dearth due to pump failure: in the ATLAS trial myocardial infarction was reported as the mode of death in 28% of autopsied patients but only in 4% of non-autopsied patients. In the same study acute coronary pathologies were observed in 54% of patients classified as dying of sudden cardiac death and in 32% of patients classified as dying of pump failure. In the OPTIMAAL trial acute myocardial infarction was autopsy-supported diagnosed in 57% of patients with ischemic cardiomyopathy, but was previously clinically diagnosed in only 16% of the patients later autopsied. Again, acute myocardial infarction was observed in 55% of patients classified as dying of sudden cardiac death and even in 81% of patients classified as dying of pump failure.

In accordance to these analyses acute myocardial infarction was also barely recognized as cause of death in our patient population (16%—29 of 180 patients who died). By hypothetically transforming the autopsy results of the OPTIMAAL trial on our patients additional 5% (8 out of 14 deaths classified as sudden) and 45% (69 out of 85 deaths classified as progressive heart failure) of cases may actually have died from MI, resulting in overall 69% of patients who died from MI^12^. Our analyses show, that patients with more severe HF were characterized by a more distinct activation of the coagulation system (Table 2). Within this patient group patients treated with PCI using first-generation DES had a higher risk of death than those treated with newer-generation DES. Based on the literature described we suggest that, this higher risk of death is caused by higher rates of coronary events as triggered by the use of the more thrombotic first-generation DES.

### Limitations

In our analysis Cox proportional hazards regression models were applied to define independent markers of death and to adjust for potential confounders. However, the results of our observational study could be biased by unmeasured confounding. As this study was not a randomized controlled trial patients with first- and newer-generation DES can not be directly compared: the selection of treatment was mainly influenced by time of treatment. It is conceivable that differences in heart failure therapy could have had an impact on the outcome of the two patient groups. However, in the univariate analysis there was no difference in the treatment with neurohumoral antagonists and with implanted cardioverter defibrillators. Accordingly, the results of this analysis have to be interpreted with caution, and are only hypothesis-generating.

## Conclusions

In patients with ischemic cardiomyopathy increased NT-proBNP levels predict a higher risk of death after PCI with first- compared to newer-generation DES. Regarding the improved technology of the newer-generation DES, the reduction of the stent strut thickness may be of particular importance, as thicker struts increase the risk of stent-thrombosis^1^. Especially the recently focused bioresorbable stents show thicker stent struts than those of newer generation DES. This should be kept in mind when treating heart failure patients with CAD by PCI. Further studies should evaluate if bioresorbable stents with thicker stent struts are safe in patients with heart failure.

## Data Availability

The data that support the findings of this study are available from the corresponding author on request.
